# Bergamot Byproducts: A Sustainable Source to Counteract Inflammation

**DOI:** 10.3390/nu16020259

**Published:** 2024-01-15

**Authors:** Caterina Russo, Giovanni Enrico Lombardo, Giuseppe Bruschetta, Antonio Rapisarda, Alessandro Maugeri, Michele Navarra

**Affiliations:** 1 Department of Chemical, Biological, Pharmaceutical and Environmental Sciences, University of Messina, Viale F. Stagno d’Alcontres 31, 98166 Messina, Italy; carusso@unime.it (C.R.); gelombardo@unime.it (G.E.L.); antonio.rapisarda@unime.it (A.R.); mnavarra@unime.it (M.N.); 2 Department of Veterinary Sciences, University of Messina, Viale G. Palatucci, 98168 Messina, Italy; giuseppe.bruschetta@unime.it

**Keywords:** inflammation, *Citrus bergamia*, bergamot, polyphenols, byproducts, *Citrus* fruits, natural products, flavonoids, waste valorization

## Abstract

Chronic inflammation is the result of an acute inflammatory response that fails to eliminate the pathogenic agent or heal the tissue injury. The consequence of this failure lays the foundations to the onset of several chronic ailments, including skin disorders, respiratory and neurodegenerative diseases, metabolic syndrome, and, eventually, cancer. In this context, the long-term use of synthetic anti-inflammatory drugs to treat chronic illnesses cannot be tolerated by patients owing to the severe side effects. Based on this, the need for novel agents endowed with anti-inflammatory effects prompted to search potential candidates also within the plant kingdom, being recognized as a source of molecules currently employed in several therapeutical areas. Indeed, the ever-growing evidence on the anti-inflammatory properties of dietary polyphenols traced the route towards the study of flavonoid-rich sources, such as *Citrus bergamia* (bergamot) and its derivatives. Interestingly, the recent paradigm of the circular economy has promoted the valorization of *Citrus* fruit waste and, in regard to bergamot, it brought to light new evidence corroborating the anti-inflammatory potential of bergamot byproducts, thus increasing the scientific knowledge in this field. Therefore, this review aims to gather the latest literature supporting the beneficial role of both bergamot derivatives and waste products in different models of inflammatory-based diseases, thus highlighting the great potentiality of a waste re-evaluation perspective.

## 1. Introduction

The earliest records about plants having health benefits date back at least 5000 years ago, to the age of the Sumerians [1]. For thousands of years, plant kingdom represented the basis of traditional medicine and even today continues to be explored for the numerous remedies that it provides. The renewed interest of the scientific community towards natural products dramatically enhanced in the last two decades and resulted in the discovery of new plant sources and their byproducts for the prevention and treatment of several diseases, including inflammatory-based ones [2]. This is the case for *Citrus bergamia* Risso (bergamot), an endemic plant of the southern coast of the Calabria region (Italy), to which we have focused our studies for over a decade, thus documenting relevant pharmacological activities [3,4,5]. 

Chronic and age-related diseases, including metabolic, autoimmune, cardiovascular and neurodegenerative ones, are primarily associated with a status of systemic and low-grade chronic inflammation, as well as oxidative stress. In particular, a dysregulation of the cytokine network along with a redox imbalance and DNA damage leads to the activation of a cytosolic protein complex called inflammasome. This complex is known to stimulate nuclear factor kappa B (NF-κB), mitogen-activated protein kinases (MAPKs), Janus kinase (JAK) signal transducer and the activator of transcription (STAT) signaling pathways, thus triggering the interleukin (IL)–1β-mediated inflammatory cascade [6]. Therefore, the resolution of this process constitutes a strategy to improve the symptoms and quality of life of patients with chronic inflammatory diseases. However, the severe side effects and/or high costs of conventional therapies, consisting of the use of corticosteroids, nonsteroidal anti-inflammatory drugs (NSAIDs) or biologic drugs, impair their tolerability and the compliance of patients. Thereby, the route towards natural products has led to the evaluation of the potential anti-inflammatory effects of bioactive molecules and phytocomplexes, also obtained from the waste of food processing [7]. 

In the last years, significant advancements have been reached on the study of the anti-inflammatory properties of bergamot derivatives and byproducts, observing its beneficial effects in different models of disease [8]. On this basis, we aimed at gathering the recent literature on the anti-inflammatory potential of bergamot, with the purpose to shed light on its role for each inflammatory disease.

## 2. Inflammatory Process

Inflammation is an immune response to injurious stimuli, including pathogens, damaged cells and toxins, which can cause tissue or organ damage. It is a defense mechanism triggered with the aim of removing the harmful agent and thus initiating a healing process leading to restoration of tissue or organ homeostasis. At the local level, inflammation is characterized by redness, heat, swelling, pain and loss of tissue function, occurring as result of an increase in vascular permeability, leukocyte recruitment and the accumulation and release of inflammatory mediators (such as cytokines, chemokines and complement proteins). Although the etiology of inflammation may be infectious (caused by bacteria, viruses or other microorganisms) or non-infectious (caused by physical, chemical and biological stimuli), the processing of the inflammatory response involves common events [9].

The first event consists of the recognition by pattern receptors, located on the surface of immune cells and named pattern-recognition receptors (PRRs), of specific signals, which are released during tissue cell damage. These signals, known as pathogen-associated molecular patterns (PAMPs), include microbial structures, which account for the activation of an infectious inflammatory response; on the contrary, other signals, known as danger-associated molecular patterns (DAMPs), including endogenous biomolecules, are responsible for the triggering of non-infectious inflammatory responses. Among PRRs, the most known class is that of Toll-like receptors (TLRs) [10]. Of note, signal transmission between PAMPs/DAMPs and TLRs, such as TLR4, is mediated by myeloid differentiation factor-88 (MyD88) and culminates in the nuclear translocation of downstream transcription factors, such as NF-κB, the activator protein 1 (AP-1) or the interferon regulatory factor 3 (IRF3) [11,12]. 

The second event of the inflammatory response reflects the activation of specific pathways of inflammation, mainly NF-κB, MAPKs and JAK-STAT pathways. In the presence of an inflammatory stimulus, the PRRs activate the IκB-kinase (IKK), which in turn promotes the activation of NF-κB via phosphorylation and degradation of the inhibitor of nuclear factor kappa B (IκB). The activation of NF-κB implies the release of pro-inflammatory cytokines (such as IL-1β, IL-6, IL-8, IL-12 or tumor necrosis factor-TNF-α), chemokines (monocyte chemoattractant protein-MCP-1 or macrophage-inflammatory protein-MIP-2) and immune cells in the focus of inflammation [13]. A phlogosis state also involves the activation of extracellular signal-regulated kinase (ERK), c-Jun *N*-terminal kinase (JNK) and p38 MAP kinases [14]. The JAK-STAT signaling pathway converts the inflammatory signal into a transcriptional response, thus regulating the expression of a variety of inflammatory genes such as cytokines, chemokines, interferons, colony stimulating factors (CSFs) or transforming growth factors (TGFs) [15].

The third event characterizing the inflammatory process is the release of specific markers of inflammation. They mainly include chemokines and inflammatory cytokines, subdivided into interleukins (such as IL-1β, IL-6 and IL-10), tumor necrosis factors (TNFs, such as TNF-α), interferons (IFNs, such as IFN-γ), colony stimulating factors (CSFs, such as granulocyte macrophage (GM)-CSF), and transforming growth factors (TGFs, such as TGF-β). The release of these molecules by immune cells is functional at recruiting leukocytes to the site of inflammation, via establishing a complex network of interactions [16]. In addition, high production of oxidative biomarkers, such as reactive oxygen species (ROS), 8-oxo-2′-deoxyguanosine (8-oxo-dG) as well as high levels of malondialdehyde (MDA) may represent the prelude to an inflammatory response or oxidative disorders [17]. The inflammatory process also implies the alteration of *C*-reactive protein (CRP) levels, the impaired activity of nuclear factor erythroid-2-related factor 2 (Nrf2) and several enzymes, such as inducible nitric oxide synthase (iNOS), prostaglandin-endoperoxide synthase (PTGS)-2, known as cyclooxygenase (COX)-2, NADPH oxidase (NOX), superoxide dismutase (SOD), catalase (CAT), glutathione peroxidase (GPx) and high mobility group box 1 (HMGB1) [18,19,20].

The fourth event determining phlogosis is the recruitment of specific cells at the site of inflammation. Based on the stage of the ongoing inflammatory process, neutrophils, monocytes/macrophages, lymphocytes, mast cells and platelets are involved [21]. All these cells, in turn, amplify the acute inflammatory response by releasing inflammatory mediators at local levels.

However, it is essential that inflammation resolves in a timely and controlled way [22]. This is because uncontrolled acute inflammation can evolve into chronic inflammation, becoming the cause of several chronic inflammatory diseases.

## 3. Main Pathological Conditions Linked to Inflammation

Inflammation represents the pathogenesis of many chronic diseases by involving common mediators and pathways. Indeed, specific markers can be predictive of some pathologies and functional to define their diagnosis, prognosis and treatment. A high production of inflammatory cytokines, an abnormal activation of inflammatory enzymes and/or proteins along with immune alterations and severe conditions of oxidative stress can cause tissue damage and organ failure [9].

Various types of skin disorders can exhibit an inflammatory etiology. This is the case of acne vulgaris, a dermatological disease caused by altered keratinization, androgen-induced sebum, inflammation and *Propionibacterium acne* colonization [23]. Patients with atopic dermatitis, driven by the JAK pathway, are highly exposed to *Staphylococcus aureus* that contributes to the generation of an inflammatory state, where pro-inflammatory cytokines (IL-13, 31 and 33) are massively released [24]. Clinically, psoriasis can exhibit highly inflammatory, pustular or erythrodermic forms. In this case timely treatment is necessary because of the possible increase in inflammation, mediated by TNF-α, IL-17, IL-23 cytokines, throughout the organism with consequent systemic impairment of other organs [25]. Like psoriasis, rheumatoid arthritis represents an immune-based inflammatory disease with high levels for TNF-α and IL-6 cytokines [6].

Chronic respiratory diseases can be caused from unresolved acute inflammation, which in turn provokes pulmonary fibrosis and impaired gas exchange through the airways. This occurs in pathologies such as cystic fibrosis, acute respiratory distress syndrome, asthma and chronic obstructive pulmonary disease (COPD) [26]. In asthma, the activation of the MAPKs pathway stimulates immune cells to release pro-inflammatory factors as well as induces goblet cells to produce mucus, causing hyperresponsiveness and obstruction of airways [27]. The JAK-STAT pathway is also responsible for the activation of inflammatory processes leading to the impairment of airways [28]. The COX-2 enzyme is implied in spasms of airways via the production of prostaglandin E2 (PGE2) [29]. In the same context, the peroxisome proliferator-activated receptors (PPARs) have been shown to influence the gene expression of lipid mediators of inflammation, such as leukotrienes [30,31].

Inflammation and oxidative stress are pillars of diseases like obesity [32]. In particular, overnutrition causes saturation of fat deposits and malfunction of tissue adipose, which becomes hypertrophied. Here, the fat accumulation triggers a pro-inflammatory state, promoting the recruitment of inflammatory cells, with release of TNF-α and IL-6 cytokines and MCP-1 chemokines. Both cytokines and chemokines impair the responsiveness of adipose tissue to insulin, meaning adipocytes are unable to capture fatty acids in excess in circulation. As a result, circulating fatty acids are accumulated in the liver, thus triggering the process of steatosis [33]. Then, the excessive inflammation which affects hepatic parenchyma increases the risk of development of non-alcoholic fatty liver disease (NAFLD) [34]. Moreover, the liver also represents a target for infectious inflammatory diseases like viral hepatitis [35].

Inevitably, the onset of metabolic syndrome, a cluster of obesity, insulin resistance and cardiovascular disorders, underlies an inflammatory pathogenesis. In particular, the adipocytes secrete MCP-1, TNF-α and IL-6, which promote the infiltration of macrophages into adipose tissue [36]. In turn, TNF-α activates the JNK and IKK kinases, which phosphorylate the insulin receptor substrate (IRS)-1 and then impair the insulin-induced uptake of glucose, resulting in insulin resistance [37]. At the same time, high levels of TNF-α as well as IL-6 amplify the inflammatory response, through the activation of NF-κB [38]. Consequently, NF-κB increases the release of chemokines and cytokines, the recruitment of inflammatory cells and the expression of adhesion molecules on endothelial cells (such as vascular cell adhesion molecule-VCAM-1, intracellular adhesion molecule-ICAM-1 and E-selectin), leading to formation of foam cells and then to atherosclerosis [39].

Several inflammatory mediators come into play during the atherosclerotic process, where the activation of nucleotide-binding domain leucine-rich repeat protein 3 (NLRP3) inflammasome by oxidized low-density lipoproteins (LDLs) stimulates the IL-1β signaling pathway [40]. Moreover, inflammatory biomarkers such as CRP and TNF-α seem to be predictors for type 2 diabetes and some cardiovascular events [41,42]. 

Again, the complex of inflammatory bowel diseases, known as IBD, including ulcerative colitis (UC) and Crohn disease (CD), is caused by chronic inflammation of the gastrointestinal tract [43]. The etiology of these conditions is still elusive, though it is thought that a compromised immune system, due to a hereditary component, can possibly react improperly to environmental stimuli such as viruses or bacteria, resulting in gastrointestinal inflammation [44]. Their development is cytokine-mediated, in particular by IL-17, IL-12 and IL-23 cytokines [45]. Other cytokines also involved are TNF-α, IL-1β and IL-6, along with NF-κB factor, which plays a key role in the pathogenesis of these diseases by stimulating the production of the COX-2 enzyme [46].

Kidneys are organs frequently exposed to damage by toxicants since their function consists of maintaining cell homeostasis and reaching a balance between the extracellular and intracellular environment by removing toxic substances from the organism. This also reflects on the adrenal glands, which respond to stress via altering neuroendocrine equilibrium [47,48]. In the kidneys, interstitial and tubular inflammation is recurrent in cases of acute and chronic renal disease, or glomerulonephritis may also occur. Following an inflammatory stimulus (DAMPS, PAMPS, high levels of glucose, etc.), epithelial renal cells promote the release of cytokines (TNF-α and IL-1β) and leukocyte infiltration, resulting in the activation of the NF-κB and MAPKs pathways [49]. 

Inflammation and oxidative stress have also been established as the major causes of neurodegeneration in the brain [50]. In Parkinson’s and Alzheimer’s diseases, specific central events occur, including the activation of the NLRP3 inflammasome and NF-κB pathway as well as of astrocytes and microglia, the main effectors of neuroinflammation. These cells release IL-1β, IL-6, TNF-α and IL-18 pro-inflammatory cytokines, accounting for the neuronal dysfunction and death [51]. 

Finally, prolonged inflammation can be pathological and lead to a malignant progression. In particular, bacterial and viral infections evolving chronic inflammation can represent a contributory factor for oncogenesis [52].

Given the key role of inflammation in several diseases, the unceasing research for valuable anti-inflammatory agents appears to still be an open challenge. 

## 4. Bergamot Derivatives and Byproducts

Several studies have shed light on the value of *Citrus* fruits, encouraging their cultivation and widespread nutraceutical interest [53,54]. This is the case of bergamot, for which 90% of the global production occurs along the Ionian coast of the province of Reggio Calabria (Italy), finding a favorable microclimate for cultivation. Here, bergamot fruit is called “green gold” due to its economic value and its greenish color, which changes to straw yellow when it ripens. The remaining 5% of bergamot production come from Greece, Morocco, Turkey, Iran, Ivory Coast, Argentina and Brazil. 

*Citrus bergamia* Risso, bergamot, is considered a cross between *Citrus aurantium* L. (sour orange) and *Citrus aurantiifolia* (Christm.) Swingle (lime) or *Citrus limon* L. (limon), although its botanical and geographical origins still remain uncertain [55]. Bergamot fruit, from the Turkish “beg armūdi” meaning “prince’s pear”, is a pyriform hesperidium with a weight of between 80 and 200 g. It is characterized by a thin epicarp (flavedo), covered with waxes, rich in schizolysigenous oil glands, accounting for the distinctive aromatic oil, colored from light yellow to orange or green, with various shades depending on the degree of ripeness of the fruit, and consists of small, dense collenchyma cells, which contain chromoplasts. Internally, it has a white, spongy and dry mesocarp (albedo), consisting of loosely connected, colorless cells and numerous air spaces in it, hence conferring a white color to this part of the hesperidium. Below the albedo is the endocarp, divided into septa resulting from the modification of the carpel leaves arranged with radial symmetry around the axis. The endocarp is relatively thin and is made up of very elongated, thick-walled epidermal cells, from which spindle-shaped pedunculated vesicles rich in juice develop; citric acid, together with a complex mix of other acids and sugars, is present in the juice vesicles, which gives the characteristic flavor to fruit [56]. The fruit contains white ovoid seeds. However, the molecular composition of each fruit can be influenced by harvesting time (from October to March), type of cultivar (Femminello, Castagnaro or Fantastico) and its degree of ripeness [57].

The fame of bergamot is linked first and foremost to its essential oil (namely BEO), which is isolated from the peel by cold-pressing or steam distillation procedures. BEO consists of a volatile fraction (93–96%), constituted by monoterpenes such as limonene, linalool, linalyl acetate, and a non-volatile fraction (4–7%), including coumarins and psoralens, such as bergamottin, bergapten and citropten [58]. Its use is well-established in the cosmetic industry as the base of many perfumes and fragrances and in the food industry where it is employed as a flavoring ingredient in the preparations of teas, liqueurs and confectionery products. Noteworthy is the role of BEO as an antiseptic due its antimicrobial activity [59]. Moreover, in vitro experiments documented the potential anticancerogenic effect of BEO [60,61].

**Figure 1:**
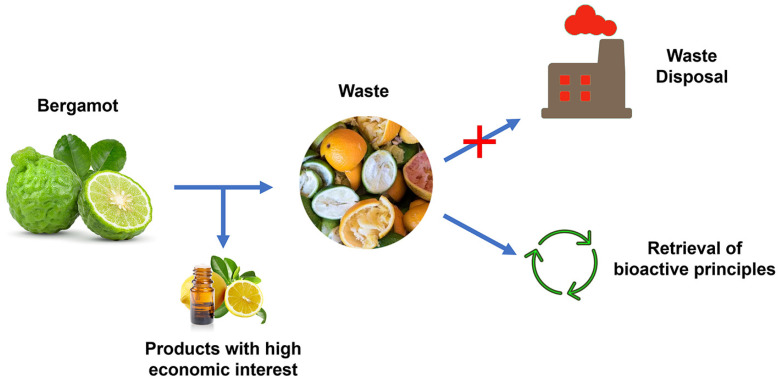
Path of bergamot processing from obtaining products with high economic interest to waste disposal. The waste valorization represents a great strategy both for recovery of compounds endowed with pharmacological properties and reduction of environmental disposal costs.

Approximately 50–65% of the waste generated during the BEO extraction process (peel, albedo and juice) must be properly handled by the manufacturing industry. If this does not occur, the large quantity of waste created yearly might be a serious environmental hazard. As a result, recovering phytochemicals endowed with high biological value from bergamot waste may provide a viable sustainable option [62] (Figure 1).

In this frame, bergamot juice (BJ), obtained by pressing of the remaining fruit pulp, has long been considered a waste product for the essence industry. Nevertheless, in the last ten years, BJ has attracted the attention of the scientific community, being recognized as a source of countless bioactive compounds. The chemical composition of BJ mainly consists of a high content of flavonoids, including naringin, neohesperidin, neoeriocitrin, melitidin, brutieridin and in diosmin, and traces of poncirin and rhoifolin [63], whose biological activity is widely acknowledged [64]. In addition, it is rich in vitamins, minerals, organic acids, sugars, proteins, dietary fiber, pectin and phosphates. 

**Figure 2:**
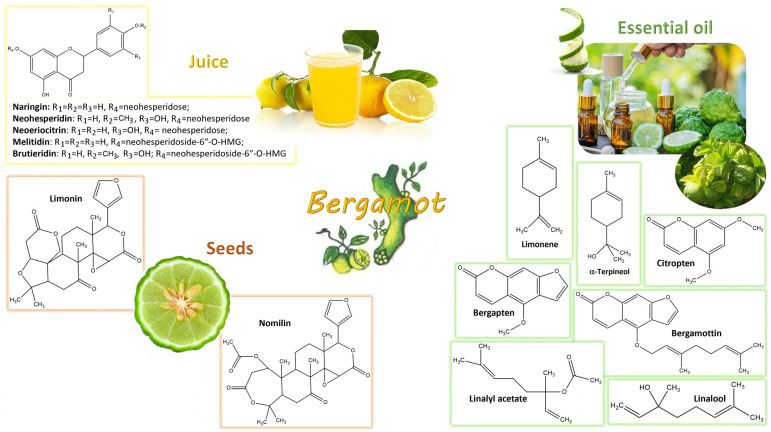
Main chemical composition of bergamot derivatives and byproducts. Bergamot juice is mostly characterized by flavonoids (i.e., naringin, neohesperidin, neoeriocitrin, melitidin and brutieridin), whereas bergamot seeds are characterized by limonoids (i.e., limonin and nomilin). Bergamot leaf oil is rich in monoterpenes (i.e., linalool, linalyl acetate and α-terpineol) and bergamot essential oil obtained from fruit peel is mainly composed of monoterpenes (i.e., limonene, linalool and linalyl acetate), coumarins (i.e., citropten) and psoralens (i.e., bergamottin and bergapten).

Finally, the seeds and leaves of bergamot have been recently revaluated. An extract obtained from bergamot seeds, including nomilin and limonin as major components, was shown to possess antiretroviral activity against human T-lymphotropic virus type 1 (HTLV-1) infection [65]. Potentially bioactive compounds such as linalyl acetate, linalool, and α-terpineol were also identified in bergamot leaf oil [66]. Figure 2 depicts chemical structures of the main bioactive components characterizing bergamot derivatives and byproducts.

## 5. Anti-Inflammatory Activity of Bergamot Derivatives and Byproducts

### 5.1. Bergamot Derivatives and Byproducts against Acute Inflammation

Bergamot was shown to be able to exert anti-inflammatory effects on experimental models of acute inflammation. In an in vitro model, a flavonoid-rich extract of bergamot juice (BJe) attenuated the inflammatory response in the leukemic monocyte THP-1 exposed to lipopolysaccharide (LPS) via the activation of the adenosine monophosphate-activated protein kinase (AMPK)/sirtuin (SIRT)1 axis [67]. Consistently, the same extract also unveiled its anti-inflammatory potential in an in vivo model by improving LPS-induced gingival inflammation in rats. Here, BJe reduced the nuclear translocation of NF-κB, the expression of TNF-α and IL-1β cytokines and ICAM and *P*-selectin adhesion molecules, as well as the myeloperoxidase activity at the gingival tissue level [68]. Similar to BJe, a BEO fraction deprived of furocoumarins (BEO-FF) produced anti-inflammatory effects on an animal model of acute inflammation. In this latter case, BEO-FF significantly reduced the carrageenan-induced paw edema in rats, decreasing levels of IL-1β, IL-6 and TNF-α in the paw homogenates, and those of nitrite/nitrate and PGE2 in exudates [69]. In Table 1, evidence on the effects of bergamot derivatives and byproducts against acute inflammation is reported.

### 5.2. Bergamot Derivatives and Byproducts in Inflammatory-Based Respiratory Ailments

Regarding chronic respiratory diseases, BEO exhibited relevant anti-asthmatic effects both in vitro and in vivo models. In the first case, BEO was able to suppress the release of inflammatory cytokines (IL-6, IL-1β and TNF-α) and inhibited their gene expression (*Il6*, *Il1b* and *Tnf-alpha*) in MH-S cells exposed to LPS. Here, it also hampered the activation of MAPKs and JAK-STAT signaling pathways and lowered the levels for key genes PTGS2 (*Ptgs2*) and PPARα (*Ppara*) [70]. Again, recent insights into anti-inflammatory potential have emerged for bergapten, a psoralen present in BEO. This component exerted a negative modulation of iNOS, COX-2 and levels of PGE2 and nitric oxide (NO) and increased the release of IL-10 in RAW264.7 cells exposed to LPS [71]. Of note, both BEO and bergapten were also shown to be effective on more complex models of respiratory inflammation such as the animal ones. In this line, the inhaling of BEO by ovalbumin-induced mice improved lung inflammation and airway narrowing, via the reduction in IL-4, IL-5, IL-13 (both at gene and protein levels), IL-6, IL-1β and TNF-α levels, and inhibited collagen deposition [70]. On the other hand, bergapten was able to ameliorate the inflammatory symptoms associated with combined allergic rhinitis and asthma syndrome (CARAS) in a mouse model. Indeed, the administration of bergapten limited the release of pro-inflammatory cytokines, the activation of STAT3 and MAPKs signaling pathways, the infiltration of inflammatory cells and collagen deposition at the level of nasal mucosa and lung tissue [72]. Table 2 shows the effects of BEO on inflammatory-based respiratory ailments.

### 5.3. Bergamot Derivatives and Byproducts against Metabolic Syndrome

Inflammation precedes and aggravates the cluster of metabolic and cardiovascular disorders known as “metabolic syndrome” [73]. In this context, several studies documented the protective role of bergamot derivatives and byproducts associated with their numerous biological effects, including relevant anti-inflammatory activity. In detail, two polyphenol fractions isolated from the bergamot leaf (BLPF) and fruit (BFPF) were able to inhibit the translocation and activation of NF-κB in a cellular model of IL-1α-induced inflammation, although to a different extent. Here, by comparing the analytical profile and anti-inflammatory activity of both fractions, bergamot leaves were found to be a richer source of polyphenols with respect to the fruit [74]. The same anti-inflammatory mechanism was observed for a byproduct of the BEO industry obtained by squeezing the solid residue that remains after fruit pressing, called PBJ, which reduced nuclear translocation of NF-κB in a cellular model of TNF-α-induced inflammation. PBJ proved to also be effective in an in vivo model of metabolic syndrome induced by a high-sugar and high-fat diet (HSF) [75]. Also noteworthy were the protective effects of a bergamot leaf extract (BLE) against the comorbidities associated with metabolic syndrome, such as the impaired function of skeletal muscles. In this context, BLE reduced oxidative stress and levels of TNF-α, IL-6 and IL-10 in the muscles of rats with metabolic syndrome [76]. Again, BJ was shown to alleviate hepatic steatosis in an animal model of diet-induced metabolic syndrome and cardiovascular risk via the reduction in IL-6 and TNF-α plasma levels and ROS generation [77].

It is known that the Mediterranean diet represents the gold standard among dietary patterns for its ability to counteract inflammation and related pathologies [78]. In this regard, it has been shown that the supplementation of an extract of bergamot polyphenols (BPF) in diet-induced hyperlipemic Wistar rats ameliorated several serum parameters. This has also occurred clinically in patients suffering from hyperlipemia and/or hyperglycemia, thus suggesting the BPF potential in counteracting metabolic syndrome [79].

A pilot study investigated the effects of a phytocomplex, including BJ, on untreated subjects with metabolic syndrome who followed the Mediterranean diet. It was shown that this nutraceutical complex was able to boost the beneficial effects of this type of diet through controlling metabolic syndrome parameters (total cholesterol, LDLs, high-density lipoproteins (HDLs), triglycerides (TGs) and glucose) and inflammation markers, like CRP [80]. Other causes thought to unleash metabolic syndrome are suggested to be gastric infections; indeed, it was shown that *Helicobacter pylori* is positively linked to such a condition [81]. In this field, bergamot juice effectively inhibited the viability of clinical isolates of *H. pylori*, while also synergistically boosting the effect of common antibiotics [82]. On this basis, Carresi and co-workers [83] defined the protective role of bergamot derivatives and their flavonoids in metabolic syndrome, indicating pleiotropic antioxidant, anti-inflammatory and lipid-lowering effects. Among these, the anti-inflammatory properties of bergamot derivatives and by-products have been highlighted in Table 3.

### 5.4. Bergamot Derivatives and Byproducts in Obesity and Overweight

Obesity and being overweight, which also occur in more complex conditions such as that of metabolic syndrome, are triggers for the inflammatory process. Thereby, anti-inflammatory approaches are suggested in the management of these pathologies [84]. Regarding the role of bergamot derivatives and byproducts on metabolic disorders, it was seen that a bergamot leaf extract (BLE) is able to decrease inflammation (TNF-α and IL-6 levels) and oxidative stress, acting on the adipose tissue–liver axis of obese rats, with an effect which might improve both insulin resistance and dyslipidemia conditions [85]. In the same experimental model, BLE was also studied at the hypothalamic level, where it reduced the inflammation (decreasing the production of cytokine signaling (SOCS) 3, IL-6 and TNF-α and activating the JAK2/STAT3 pathway) and oxidative stress, thus counteracting the resistance to leptin (known as the “satiety hormone”) of obese rats [86]. Clinically, a nutraceutical containing bergamot extract was shown to improve systemic inflammation, significantly reducing high-sensitivity C-reactive protein (hs-CRP) and TNF-α levels in dyslipidemic overweight subjects [87]. The beneficial role of bergamot derivatives and byproducts in counteracting obesity/overweight is collected in Table 4.

### 5.5. Bergamot Derivatives and Byproducts in Liver Diseases

It is known that the accumulation of triglycerides in hepatocytes accompanied by inflammation or cell injury is the leading cause of non-alcoholic fatty liver disease (NAFLD), which may progress into the more aggressive form of non-alcoholic steatohepatitis (NASH). In this context, BPF has been shown to decrease hepatic inflammation in rats with cafeteria diet-induced NAFLD. In detail, BPF lowered *Il-6* gene expression and increased *Il-10* mRNA levels, which were related to a reduced number of Kupffer cells and inflammatory foci in the liver [88]. Again, bergamot polyphenolic formulation (BPF99) reduced inflammation in a mouse model of NAFLD by inhibiting the activation of the JNK and p38 MAPKs pathways responsible for an overproduction of pro-inflammatory cytokines and collagen deposition, which may precede liver fibrosis [89]. Interestingly, a synergistic combination between BPF and *Cynara cardunculus* extract (CyC), namely Bergacyn, counteracted NAFLD-related symptoms in a clinical setting, inducing anti-inflammatory effects. In particular, oral administration of Bergacyn^®^ reduced oxidative stress (GPx, SOD and MDA) and inflammatory biomarkers (TNF-α), contributing to a significant improvement of vascular inflammation and NO-mediated vasodilation in patients with NAFLD and type 2 diabetes [90]. The anti-inflammatory effects described for bergamot derivatives and byproducts in the context of liver diseases are gathered in Table 5.

### 5.6. Bergamot Derivatives and Byproducts in Dyslipidemic Disorders

Of note, systemic inflammation and elevated serum cholesterol concentrations are predisposing factors for the development and progression of cardiovascular diseases, such as atherosclerosis. Interestingly, bergamot juice flavanones were shown to play a key role in restoring the endothelial functionality impaired by lipotoxic effects, a common cause of atherosclerosis. In detail, Spigoni and co-workers [91] indicated that some flavanone metabolites (i.e., hesperetin-7-O-glucuronide, hesperetin-3′-O-glucuronide, naringenin-7-O-glucuronide and naringenin-4′-O-glucuronide) were able to mitigate the stearate-induced inflammation (reducing the *Il-1b*, *Il-6*, *Il-8* and *Tnf-alpha* mRNA levels) in human pro-angiogenic cells. From a clinical point of view, a polyphenolic fraction of bergamot juice was previously shown to reduce plasma lipids and improve the lipoprotein profile in patients with moderate hyperlipidemia [4]. In this frame, Fogacci and collaborators [92] recently proved significant improvements in subjects with moderate hypercholesterolemia after dietary supplementation with a nutraceutical containing a standardized bergamot polyphenolic fraction (Eufortyn^®^ Colesterolo Plus, Scharper, Milano, Italia). This mixture reduced the levels of hs-CRP as well as the indexes of endothelial reactivity (ER) and NAFLD. Inflammation-lowering effects were also observed for another dietary supplement (BruMeChol™, Mivell, Jesi, Italia), composed of a mixture of flavonoids extracted from bergamot, olive polyphenols, plant sterols and vitamin K2, in patients with mild hypercholesterolemia. In this case, the nutraceutical combination significantly lowered circulating levels of inflammatory mediators, such as IL-6, IL-32, IL-37 and IL-38, hs-CRP, and inflamma-microRNA miR-21 and miR-146a [93]. The effects of bergamot derivatives and byproducts on dyslipidemias are described in Table 6.

### 5.7. Bergamot Derivatives and Byproducts in Renal, Gynecological and Rectal Dysfynctions

The frequent exposure of kidneys to drugs and toxic agents is the major cause of renal injury. In this regard, a bergamot extract exerted preventive effects against amikacin-induced nephrotoxicity in rats, restoring the kidney function and IL-6 serum levels that were raised by the drug [94]. Similarly, a flavonoid-rich extract of bergamot juice (BJe), alone or in association with curcumin (Cur) and resveratrol (Re), counteracted the cadmium-induced kidney damage in a murine model, restoring the antioxidant defense systems and reducing the expression of *Nos2*, *Il1b*, *Nrf2* and heme oxygenase (*Hmox)1* pro-inflammatory genes [95]. In addition, BEO, and to a minor extent BJ and an ethanol bergamot extract, was shown to be effective in gynecological disorders such as primary dysmenorrhea induced by estradiol benzoate and oxytocin in rats. These bergamot derivatives alleviated dysmenorrhea, regulating the levels of PGF_2α_ and PGE_2_ prostaglandins and the release of inflammatory mediators such as iNOS as well as activating the antioxidant defense mechanisms in the uterine tissue of rats [96]. Clinically, innovative results came from the treatment of patients affected by anitis/proctitis after treatment with a bergamot oil (known as Benebeo^®^ gel, Wellvit, Cosenza, Italia). The local administration of bergamot gel, containing hesperidin, naringenin, apigenin and eriocitrin as major components, produced anti-inflammatory effects (improvement in local bleeding and hyperemia), promoting a fast-healing process in situ [97]. The evidence of bergamot derivatives and byproducts against the abovementioned disorders is reported in Table 7.

### 5.8. Bergamot Derivatives and Byproducts in Neurological Disorders

Neuronal disorders in most cases recognize an inflammatory component at the basis of their etiology. In this regard, neuroprotective effects of *Citrus bergamia* juice extract were documented in an in vitro model of Alzheimer’s disease (AD), where BJe decreased the pro-inflammatory stimulus induced by β-amyloid on THP-1 cells [98]. 

In vivo, BEO attenuated anxiety in rats, counteracting the oxidative stress, the neuroinflammation and GABA changes induced after exposure to aluminum. As an anti-inflammatory agent, BEO significantly reduced the content of IL-6, IL-1β and TNF-α both at the hippocampal and frontal cortex levels of rats [99]. In a clinical trial, the oral administration of a bergamot polyphenolic fraction (BPF) was associated with a significant improvement in cognitive functioning, which had been impaired by ongoing inflammatory processes in schizophrenic patients [100]. The effects on neurological disorders of bergamot derivatives and byproducts are reported in Table 8.

### 5.9. Bergamot Derivatives and Byproducts in Cancer

The weak balance between the inflammatory status and unsuccessful anti-inflammatory response is at the basis of cellular degeneration, an event that may lead to serious outcomes such as cancer [53]. Within this frame, bergamot byproducts were shown to play a relevant role against several types of cancer. In vitro, BJ was able to counteract the malignant proliferation of neuroblastoma SH-SY5Y cells [101] as well as that one of human hepatocellular carcinoma HepG2 cells via targeting NF-κB, a central factor in both inflammation and cancer [102]. This is in line with the evidence of the anti-proliferative effects observed for a flavonoid-rich extract of BJ in human colon cancer HT-29 cells [103]. However, in a complex context like the tumor microenvironment, other factors come into play including the family of sirtuins. Interestingly, the antileukemic effects observed for BJe and some flavanones it contains on leukemic THP-1 cells were mediated by the inhibition of the SIRT2 enzyme [104,105]. Anticancer effects induced by bergamot derivatives were also observed in murine models [106]. In particular, anti-inflammatory mechanisms were related to the potentiality of BJe to prevent colorectal carcinogenesis in Pirc rats (F344/NTac-Apc^am1137^). Here, a significant reduction in colon tumors and mucin-depleted foci was recorded in BJe-treated groups. This effect was ascribed to the extract’s capacity to inhibit apoptosis and decrease the expression of *Ptgs2*, *iNos*, *Il-1b*, *Il-6*, *Il-10* and Arginase *(Arg)1* pro-inflammatory biomarkers [107]. The beneficial role of bergamot derivatives and byproducts against cancerous pathways are presented in Table 9.

### 5.10. Bergamot Derivatives and Byproducts in Skin Disorders

In the field of skin diseases, BEO with its major components, namely limonene, linalyl acetate and linalool, showed significant antiproliferative activity on a pre-inflamed human dermal system, consisting of primary fibroblasts stimulated with a mixture of IL-1β, TNF-α, IFN-γ, basic fibroblast growth factor (bFGF), epidermal growth factor (EGF) and platelet-derived growth factor (PDGF) to simulate chronic inflammation. In the same model, BEO significantly inhibited the expression of proteins related to inflammation (MCP-1, VCAM-1, ICAM-1, interferon gamma-induced protein (IP-10), interferon-inducible T cell alpha chemoattractant (I-TAC) and monokine induced by gamma interferon (MIG)) and tissue remodeling processes (collagen I, collagen III, plasminogen activator inhibitor (PAI) 1, tissue inhibitor of metalloproteinase (TIMP) 1 and 2), thus exhibiting anti-inflammatory and wound healing properties [108]. The anti-inflammatory role of bergamot derivatives was also documented in vivo, considering that BJ improved the acne vulgaris lesions caused by an excessive secretion of androgen via reducing the release of inflammatory IL-1α, IL-6, TNF-α cytokines and the levels of matrix metalloprotease (MMP)-2 and -9 in the sebaceous gland of golden hamsters, though to a lesser extent than BEO [109]. This study is resulted in line with a previous clinical application in which a nano-phytosome (NP) formulation was developed to optimize the BEO topical delivery, experiencing synergic effects between BEO-NPs and spironolactone in acne patients [110]. Interestingly, ultradeformable nanocarriers were designed for a transdermal delivery of naturally derived compounds such as BEO and ammonium glycyrrhizinate (AG) since the proper deformability of these nanosystems facilitates movement through the skin barrier, ensuring a topical application. In particular, the co-encapsulation of BEO into AG-loaded nanoparticles preserved their degree of deformability, thus efficiently counteracting the skin inflammatory states on human volunteers [111]. In Table 10, the effects of bergamot derivatives and byproducts against skin disorders are listed.

## 6. Conclusions

Inflammation is a complex process, which affects organs and tissue with high costs for human health. Many disorders arise from an unresolved inflammatory response, evolving into chronic inflammatory pathologies. Thereby, the resolution of the phlogistic process represents the goal strategy to prevent or limit the progression of inflammatory illnesses by exerting control on the production of mediators such as the release of pro-inflammatory cytokines, the activation of specific pathways or the recruitment of infiltrating cells at the site of inflammation. 

**Figure 3:**
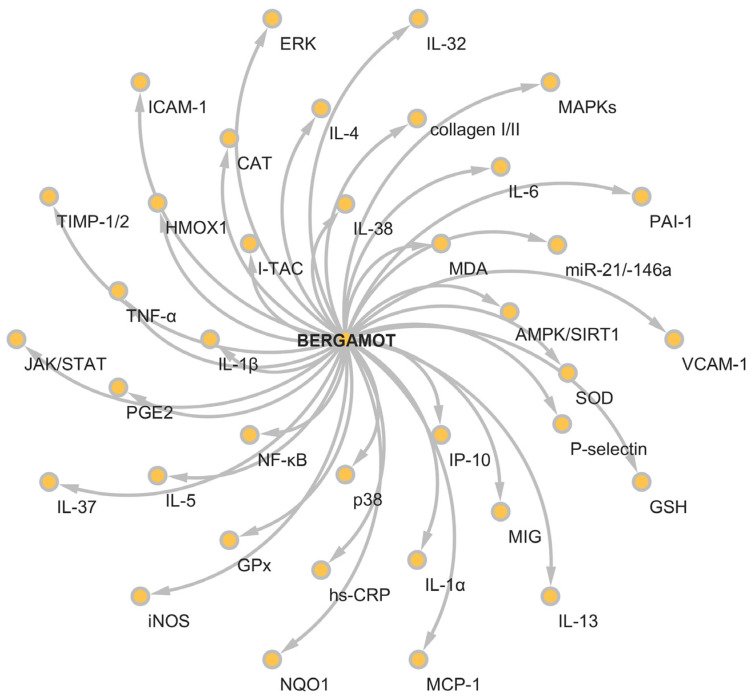
Radial map of the chemokines, enzymes and nuclear factors influenced by bergamot derivatives and byproducts.

The waste from bergamot processing has long been considered byproducts, leading not solely to a loss of valuable molecules but also increasing the environmental cost of its appropriate disposal. For over a decade, bergamot derivatives and byproducts have been demonstrated to be a relevant source of bioactive molecules, which can play a significant role in several diseases thanks to their considerable pharmacological properties, including anti-inflammatory ones (Figure 3).

Recently, new outcomes in both preclinical studies and human trials have been reached on the beneficial properties of bergamot that we summarized here, thus deciding to update our previous review on this topic. The reinforced data on the effectiveness of bergamot derivatives and byproducts on modulating the expression and release of chemokines, as well as the activity of nuclear factors and enzymes linked to the onset and progression of inflammation, contribute to the amelioration of current therapeutical strategies. Therefore, bergamot can represent a sustainable and powerful resource in the management of several inflammatory-based ailments given the robust and deep evidence in this regard.

## Figures and Tables

**Table 1:** Effects of bergamot derivatives and byproducts in acute inflammation models.

Derivatives/Byproducts	Inflammation-Related Disease	Experimental Model		Inflammatory Biomarkers	References
A flavonoid-rich extract of bergamot juice	Acute inflammation	In vitro	Leukemic monocytes THP-1 exposed to LPS	AMPK/SIRT1 axis	[67]
A flavonoid-rich extract of bergamot juice	Periodontitis	In vivo	LPS-induced gingival inflammation in rats	NF-κB, TNF-α, IL-1β, ICAM, *P*-selectin and myeloperoxidase	[68]
Bergamot essential oil fraction deprived of furocoumarins	Acute inflammation		Carrageenan-induced paw edema in rats	IL-1β, IL-6, TNF-α, nitrite/nitrate and PGE2	[69]

**Table 2:** Anti-inflammatory effects of bergamot essential oil on asthma experimental models.

Derivatives/Byproducts	Inflammation- Related Disease	Experimental Model		Inflammatory Biomarkers	References
Bergamot essential oil	Asthma	In vitro	MH-S cells exposed to LPS	IL-6 (*Il6*), IL-1β (*Il1b*), TNF-α (*Tnf-alpha*), *MAPKs1*,*3*,*8*,*14*, *Jak2*, *Stat3*, *Ptgs2* and *Ppara*	[70]
		In vivo	Ovalbumin-induced mice	IL-4 (*Il4*), IL-5 (*Il5*), IL-13 (*Il13*), IL-6, IL-1β, TNF-α and collagen deposition	

**Table 3:** Anti-inflammatory effects of bergamot derivatives and byproducts on metabolic syndrome.

Derivatives/Byproducts	Inflammation- Related Disease	Experimental Model		Inflammatory Biomarkers	References
Enriched polyphenol fraction from bergamot fruit and leaves	Metabolic syndrome	In vitro	IL-1α-induced inflammation in R3/1 NF-κB cell line	NF-κB	[74]
Powder from bergamot juice	Metabolic syndrome		TNF-α-induced inflammation in R3/1 NF-κB cell line	NF-κB	[75]
Bergamot leaf extract	Metabolic syndrome and impaired function of skeletal muscle	In vivo	Rats fed with high-sugar diet	TNF-α, IL-6 and IL-10	[76]
Bergamot juice	Metabolic syndrome		High-fat-diet-induced steatosis in rats	IL-6 and TNF-α	[77]
Nutraceutical multicompound including bergamot juice	Metabolic syndrome	Clinical trial	Untreated subjects with metabolic syndrome	CRP	[80]

**Table 4:** Anti-inflammatory effects of bergamot derivatives and byproducts against obesity and overweight.

Derivatives/Byproducts	Inflammation- Related Disease	Experimental Model		Inflammatory Biomarkers	References
Bergamot leaf extract	Obesity	In vivo	Diet-induced obese rats	TNF-α, IL-6	[85]
Bergamot leaf extract	Obesity		Obese rats fed with high-sugar and high-fat diet	TNF-α, IL-6, JAK2/STAT3 pathway and SOCS3	[86]
Nutraceutical containing a bergamot standardized flavonoid extract	Overweight	Clinical trial	Dyslipidemic overweight patients	hs-CRP and TNF-α	[87]

**Table 5:** Anti-inflammatory effects of bergamot derivatives and byproducts in liver diseases.

Derivatives/Byproducts	Inflammation- Related Disease	Experimental Model		Inflammatory Biomarkers	References
Bergamot polyphenol fraction	Non-alcoholic steatohepatitis (NASH)	In vivo	Rats exposed to a cafeteria diet	*Il-6* and *Il-10*	[88]
Bergamot polyphenolic formulation	Non-alcoholic fatty liver disease (NAFLD)		Mice fed with a Western diet	JNK, p38, procollagen I and III	[89]
Bergacyn (combination between bergamot polyphenolic fraction and *Cynara cardunculus* extract)	NAFLD	Clinical trial	Patients with NAFLD and type 2 diabetes	TNF-α	[90]

**Table 6:** Effects of bergamot derivatives and byproducts against dyslipidemias.

Derivatives/Byproducts	Inflammation-Related Disease	Experimental Model		Inflammatory Biomarkers	References
Bergamot juice flavanones (i.e., hesperetin-7-O-glucuronide, hesperetin-3′-O-glucuronide, naringenin-7-O-glucuronide and naringenin-4′-O-glucuronide)	Endothelial dysfunction	In vitro	Stearate-induced inflammation in myeloid angiogenic cells.	*Il-1b*, *Il-6*, *Il-8* and *Tnf-alpha*	[91]
Eufortyn^®^ Colesterolo Plus (a nutraceutical containing a standardized bergamot polyphenolic fraction)	Hypercholesterolemia	Clinical trial	Patients with moderate hypercholesterolemia	hs-CRP and ER	[92]
BruMeChol™ (supplement composed by a mixture of flavonoids extracted from bergamot, olive polyphenols, plant sterols and vitamin K2)	Hypercholesterolemia		Patients with mild hypercholesterolemia	IL-6, IL-32, IL-37, IL-38, hs-CRP, miR-21 and miR-146a	[93]

**Table 7:** Anti-inflammatory effects of bergamot derivatives on inflammation-related diseases affecting renal, gynecological and rectal districts.

Derivatives/Byproducts	Inflammation- Related Disease	Experimental Model		Inflammatory Biomarkers	References
Bergamot extract	Nephrotoxicity	In vivo	Amikacin-induced nephrotoxicity in rats	IL-6	[94]
A flavonoid-rich extract of bergamot juice, alone or in association with curcumin and resveratrol	Nephrotoxicity		Cadmium-induced kidney damage in a murine model	*Nos2*, *Il1b*, *Nrf2* and *Nqo1*	[95]
Bergamot essential oil, bergamot juice and ethanol extract of bergamot	Primary dysmenorrhea		Dysmenorrhea induced by estradiol benzoate and oxytocin in rats	PGF2α, PGE2 and iNOS	[96]
Benebeo^®^ gel (Bergamot oil)	Anitis/proctitis	Clinical trial	Patients with anitis/proctitis	Local bleeding and hyperemia	[97]

**Table 8:** Effects of bergamot derivatives and byproducts on inflammatory-based neurological disorders.

Derivatives/Byproducts	Inflammation- Related Disease	Experimental Model		Inflammatory Biomarkers	References
Bergamot juice extract	Alzheimer’s disease	In vitro	THP-1 cells exposed to β-amyloid	IL-6 (*Il-6*), IL-1β (*Il1b*), NF-κB, AP-1 and MAPKs pathway	[98]
Bergamot essential oil	Anxiety	In vivo	Rats exposed to aluminum	IL-6, IL-1β and TNF-α	[99]

**Table 9:** Anti-inflammatory effects of bergamot derivatives on inflammation markers related to cancer.

Derivatives/Byproducts	Inflammation- Related Disease	Experimental Model		Inflammatory Biomarkers	References
Bergamot juice	Hepatocellular carcinoma	In vitro	HepG2 cells	NF-κB	[102]
Bergamot juice extract	Colorectal cancer	In vivo	Pirc rat (F344/NTac-Apc^am1137^)	*Ptgs2*, *iNos*, *Il-1b*, *Il-6*, *Il-10* and *Arg1*	[107]

**Table 10:** Anti-inflammatory effects of bergamot derivatives and byproducts on skin disorders.

Derivatives/Byproducts	Inflammation-Related Disease	Experimental Model		Inflammatory Biomarkers	References
Bergamot essential oil	Chronic skin inflammation	In vitro	Primary fibroblasts stimulated with a mixture of IL-1β, TNF-α, IFNγ, bFGF, EGF and PDGF	MCP-1, VCAM-1, ICAM-1, IP-10, I-TAC, MIG, collagen I and III, PAI-1 and TIMP-1 and -2	[108]
Bergamot essential oil and juice	Acne vulgaris	In vivo	Administration of compound pearl acne capsules on golden hamsters	IL-1α, IL-6, TNF-α, MMP-2 and MMP-9	[109]
Encapsulation of bergamot essential oil into ammonium glycyrrhizinate-loaded nanoparticles	Skin inflammation	Clinical trial	Human volunteers subjected to methylnicotinate solution on specific skin sites	Erythema index	[111]

## Data Availability

Not applicable.
